# Proteomic profiling analysis reveals that glutathione system plays important roles responding to osmotic stress in wheat (*Triticum aestivum* L.) roots

**DOI:** 10.7717/peerj.2334

**Published:** 2016-08-17

**Authors:** Jianhui Ma, Wen Dong, Daijing Zhang, Xiaolong Gao, Lina Jiang, Yun Shao, Doudou Tong, Chunxi Li

**Affiliations:** 1 College of Life Science, Henan Normal University, Xinxiang, Henan, China; 2 China Rural Technology Development Center, Beijing, China

**Keywords:** *Triticum aestivum* L., Root, iTRAQ, Osmotic stress, Glutathione

## Abstract

Wheat is one of the most important crops in the world, and osmotic stress has become one of the main factors affecting wheat production. Understanding the mechanism of the response of wheat to osmotic stress would be greatly significant. In the present study, isobaric tag for relative and absolute quantification (iTRAQ) was used to analyze the changes of protein expression in the wheat roots exposed to different osmotic stresses. A total of 2,228 expressed proteins, including 81 differentially expressed proteins, between osmotic stress and control, were found. The comprehensive analysis of these differentially expressed proteins revealed that osmotic stress increased the variety of expressed proteins and suppressed the quantity of expressed proteins in wheat roots. Furthermore, the proteins for detoxifying and reactive oxygen species scavenging, especially the glutathione system, played important roles in maintaining organism balance in response to osmotic stress in wheat roots. Thus, the present study comprehensively describes the protein expression changes in wheat roots in response to osmotic stress, providing firmer foundation to further study the mechanism of osmotic resistance in wheat.

Osmotic stress, primarily resulting from drought or excessive salt in water, refers to insufficient water availability that limits plant growth and development (Zhu et al., 1997). Osmotic stress has become one of the major abiotic stresses affecting crop growth and production. For high-yield and high-quality production, it is imperative to improve the osmotic tolerance of crops, and some methods had been developed to alleviate osmotic stress through cultural practices, conventional breeding, exogenous regulators and molecular breeding. However, this situation has not substantially changed. To further improve osmotic tolerance, it is necessary to understand the responding mechanism of osmotic resistance in plants. Previous studies in rice, *Arabidopsis*, and other plants have been performed, including molecular cloning, transgenic studies and high throughput analyses.

Previous studies have primarily focused on molecular cloning and functional analysis of osmotic resistance genes. Many functional genes with high osmotic resistance have been identified. The water loss rate of transgenic *Arabidopsis* with *AtMYB15* over-expression was significantly reduced compared with that in wild-type under drought conditions (Ding et al., 2009). Using cDNA microarray analysis, Hu et al. (2006) observed that *SNAC1* was up-regulated in rice under drought stress and the over-expression of *SNAC1* enhanced drought tolerance in transgenic rice. Subsequently, Liu et al. (2014) achieved *SNAC1* over-expression in cotton and found that the tolerance to drought and salt stresses was significantly improved in these transgenic plants. In addition, many other genes, such as the *WRKY* (Qiu & Yu, 2009; Ma et al., 2014) transcription factor, *DREB* (Liu et al., 1998) and *AtGAMT1* (*Arabidopsis thaliana GA methyl transferase 1*) (Nir, Moshelion & Weiss, 2014; Qin & Zeevaart, 2002; Shou, Bordallo & Wang, 2004; Pasquali et al., 2008), have also been implicated in drought or salt tolerance in plants. Based on these osmotic tolerance genes, some gene regulatory networks in response to osmotic stress were also identified in plants, indicating that the mechanism of osmotic resistance is complex with multigenic control (Shinozaki, Yamaguchi-Shinozakiy & Sekiz, 2003; Valliyodan & Nguyen, 2006; Krasensky & Jonak, 2012).

In recent years, high throughput screening platforms have been rapidly developed, providing more comprehensive insights into the cellular and molecular mechanisms of the response to osmotic stress. In *Arabidopsis*, a gene microarray was performed under drought, high-salinity and cold stresses in 2008, and thousands of stress-related genes were identified, many of which had been previously reported (Matsui et al., 2008). Lenka et al. (2011) performed a transcriptome analysis of drought-tolerant and drought-sensitive rice cultivars, and found that the up-regulation of the *α*-linolenic acid metabolic pathway was closely associated with drought responses. Many studies on plant responses to osmotic stress have also been performed using RNA-seq or microarray analysis (Zheng et al., 2010; Le et al., 2012; Li et al., 2012). The results of these studies provided a platform for understanding the responses of osmotic stress at the level of gene expression.

However, proteins directly participate in the activities of organisms, and proteomic analysis has become the best strategy for studying the response of organisms to osmotic stress. Many studies have already been performed in this area. Mirzaei et al. (2012) conducted a quantitative label-free shotgun proteomic analysis using the root tissues of rice plants under four different drought treatments, and 1,487 differentially expressed proteins (DEPs) were identified. After further analysis of the DEPs, Mirzaei et al. (2012) found that the proteins involved in transport and reactive oxygen species (ROS) were highly dependent on drought signals. In cotton, Deeba et al. (2012) identified 22 drought-related proteins through two-dimensional gel electrophoresis (2-DE) analysis. In wheat, many studies on osmotic stress were also performed using 2-DE, and some osmotic-related proteins and processes were also identified (Peng et al., 2009; Caruso et al., 2009; Ge et al., 2012). However, these studies could not comprehensively describe the protein expression changes under osmotic stress due to the limitations of the technology.

The isobaric tag for relative and absolute quantification (iTRAQ) system, which uses isotope labeling combined with multidimensional liquid chromatography and tandem mass spectrometry (MS) (Fan et al., 2011), simultaneously identified and quantitatively compared proteins expressed in an organism by analyzing the peak intensities of reporter ions (Lan et al., 2011). It can provide more global information of proteins expression for proteomic analysis. In the present study, we performed proteomic analysis using iTRAQ to analyze the osmotic response in the root of wheat seedlings. A total of 2,228 proteins were identified, among which 81 proteins were found to be related to osmotic stress in wheat.

## Materials and Methods

### Plant materials and the measurement of relative water content (RWC)

Seeds of Aikang58 were sterilized using 0.1% HgCl_2_ for 7 min and washed eight times with sterile distilled water. Subsequently, the seeds were cultured in Petri dishes in a chamber under the same conditions according to Li et al. (2013). At the two-leaf stage, the wheat seedlings were transferred into Hoagland solution containing 0%, 5%, 10%, 15% and 20% PEG-6000 to simulate osmotic stress. After cultivation for 24 h, the root tissues from seedlings exposed to the five treatments were collected and frozen in −80 °C for subsequent experiments. RWC was measured according to Gao et al. (2011).

### Protein extraction

Frozen root samples were thoroughly ground into powder in liquid nitrogen. Lysis buffer (pH 8.5), containing 2 M thiourea, 7 M urea and 4% CHAPS with protease inhibitor (Sigma, USA), was added to the powder at 1:10 (w/v). The mixture was sonicated for 60 s and extracted for 30 min at room temperature. Subsequently, the mixture was centrifuged at 40,000 g for 1 h at 10 °C, and the supernatant was transferred to a 50 mL tube containing four volumes of 10% (w/v) TAC/acetone. After mixing, the mixture was stored at −20 °C overnight, and the supernatant was removed after centrifugation at 40,000 g for 10 min at 4 °C. The protein was washed three times with acetone and then dried through lyophilization to form a protein powder, and suspended in lysis buffer (2 M thiourea, 7 M urea and 4% CHAPS). The protein concentration was determined using the Bradford assay with BSA as a standard. The remaining samples were stored at −80 °C until further use.

### Trypsin digestion and iTRAQ labeling

**Figure 1:**
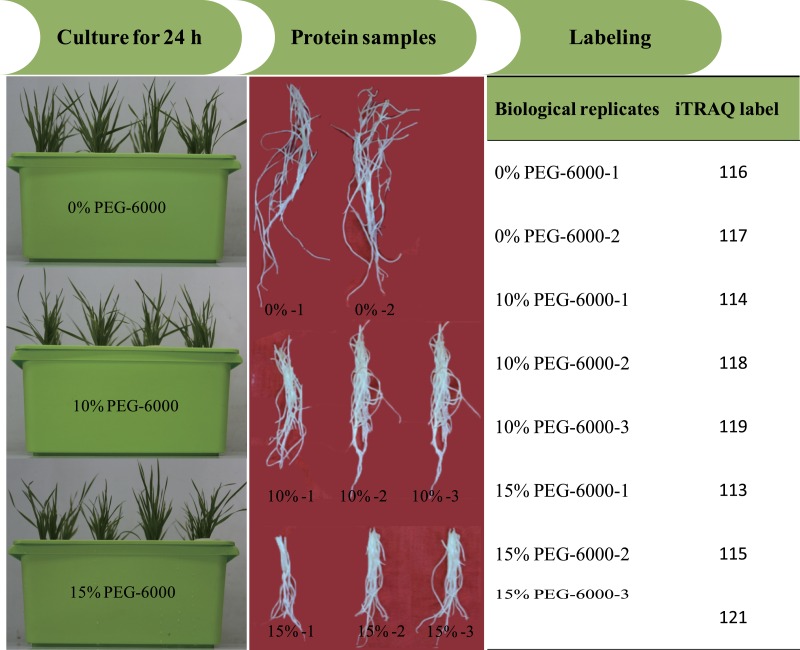
The illustration of the experimental design.

All reagents and buffers for iTRAQ labeling and cleaning were purchased from Applied Biosystems (Foster City, CA, USA). iTRAQ labeling was performed according to the manufacturer’s instructions. The proteins were dissolved, denatured, alkylated and digested with trypsin at 37 °C overnight. And 100 µg of the digestion product were thawed and reconstituted in 150 µL of isopropanol, and subsequently labelled with iTRAQ reagent (Applied Biosystems). The iTRAQ experiment just contained two experiment settings of four-plex and eight-plex, which could only analyze four or eight samples once time respectively. In previous studies, researchers had performed the eight-plex iTRAQ experiments using the setting of 3:3:2 and 3:2 (Longworth et al., 2012; Ge et al., 2014). In the present study, the experiment setting of 3:3:2 (eight-plex) was selected for this analysis. The three biological replicates of roots exposed to 15% PEG-6000 treatment were labeled with 113, 115 and 121 tags, the three biological replicates of roots exposed to 10% PEG-6000 treatment were labeled with 114, 118 and 119 tags, and the two biological replicates of control (0% PEG-6000) were labeled with 116 and 117 tags (Fig. 1). Subsequently, the labeled samples were pooled in equal ratios. The labeled peptide mixture was dissolved in 100 µL mobile of phase A (2% (v/v) acetonitrile, 98% (v/v) ddH2O, pH 10) and subsequently centrifuged at 14,000 g for 20 min. The supernatant was carefully collected and further loaded onto the column for stepwise elution through the injection of mobile phase B (98% acetonitrile, 2% ddH2O, pH 10) with a 700 µl/min flow rate. The fractions were eluted (1.8 min each) and collected using step gradients of mobile phase B.

### Analysis using Q-Exactive mass spectrometer

The fractionated peptides were analyzed using a Q-Exactive mass spectrometer (Thermo Fisher Scientific, Waltham, MA, USA) fitted with a nano-liquid chromatography system (Thermo Scientific EASY-nLC 1000 System). A binary solvent system comprising 99.9% H2O, 0.1% formic acid (phase A) and 99.9% ACN, 0.1% formic acid (phase B) were used to elute the peptides. The following linear gradient was used: 4–8% B in 5 min, 8–35% B in 35 min, 35–90% B in 5 min, washed at 95% B for 6 min, and equilibrated with 4% B for 8 min at a 350 nL/min flow rate. The eluent was further introduced to a Q-Exactive mass spectrometer via an EASY-Spray ion source. The following source ionization parameters were used: 2.1 kV spray voltage, capillary temperature 250 °C and 100 V declustering potential.

A Top 20 data-dependent mode with automatic switching between MS and MS/MS was used in mass spectrometer. Full-scan MS mode (350–1,800 m/z) was performed at a resolution of 70,000 with 1 × 10^6^ ions automatic gain control (AGC) target and a maximum ion transfer (IT) of 60 ms. The precursor ions were fragmented using high-energy collisional dissociation (HCD) and subjected to MS/MS scans with the following parameters: 17,500 resolution, AGC with 5 × 10^6^ ions, maximum IT with 70 ms, 5,000 intensity threshold and 29% normalized collision energy.

### Sequence database searching and data analysis

Mascot 2.2 (Matrix Science, London, UK) and Proteome Discoverer 1.4 (Thermo Electron, San Jose, CA) were used for processing the raw data of MS/MS spectra and completing database search and a quantitative analysis against a non-redundant protein database of hexaploid wheat genome, which had been generated by Mayer et al. (2014) and provided as File S1. For database searching, the following parameters were used: trypsin enzyme, two missed cleavages at maximum, 20 ppm of peptide mass tolerance, 0.1 Da of fragment mass tolerance, carbamidomethylation of cysteine as fixed modification, methionine oxidation and iTRAQ 8 plex labels at the N-termini and at lysine side chains as dynamic modification. For protein identification, only peptides with significant scores (iron score ≥ 35) at 99% confidence interval were used, and 2,228 proteins were finally got, of which 1,391 proteins with two or more peptides were considered for further analysis. The protein fold-change was obtained based on the quantity comparison between each treatment sample and the average level of control. For statistical analysis, the average fold-change ≥ 95% confidence interval and *P*-values ≤ 0.05, which was got by the *t*-test with different repeat times in two groups, were considered significant. The sequence data of the DEPs was searched against the UniProt database for protein function, and the BlastKOALA website (http://www.kegg.jp/blastkoala/) was used for the KEGG analysis with an *E*-value of 1 × 10^−5^.

The mass spectrometry data have been deposited to the iProx database with the accession number: IPX00075800.

### Phylogenetic analysis of glutathione S-transferases (GSTs)

Multiple amino acid sequence alignment of GSTs was performed using ClastalW. An unrooted phylogenetic tree of these GST protein sequences was constructed using the neighbor-joining method with MEGA 5.10 software, and a bootstrap analysis with 1,000 replicates was performed to assess the significance of each node.

## Results and Discussion

### The effects of osmotic stress on wheat seedlings

**Figure 2:**
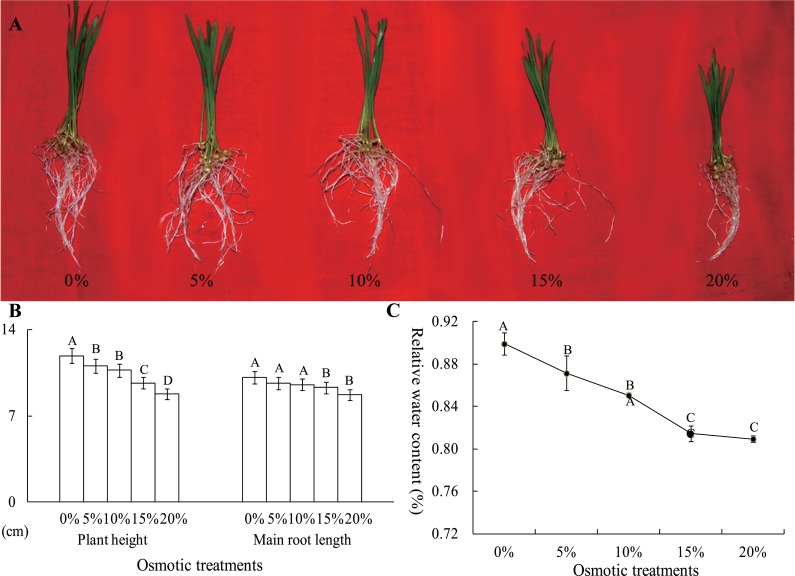
The plant height, main root length (B) and RWC of wheat seedling at the two-leaf stage, which were exposed to five osmotic stresses, were measured to assess the effects of osmotic. The data was analyzed by one-way ANOVA analysis, and the LSD method was used for multiple comparisons. The significant difference is represented by capital letters at 0.01 level.

To analyze the effects of osmotic stress, five different osmotic treatments (0%, 5%, 10%, 15% and 20% PEG-6000) were performed on wheat seedlings at the two-leaf stage. After cultivation for 24 h, the plant height and main root length were severely restrained by osmotic stress, declining to 8.76 and 8.74 cm from 11.88 and 10.13 cm, respectively (Figs. 2A and 2B). The RWC of whole plants was measured, and this value was significantly different between the control and osmotic treatment samples. The RWC was 89.92% after a 0% PEG-6000 treatment and decreased to 81.44% after a 15% PEG-6000 treatment. However, when the PEG-6000 treatment increased to 20% from 15%, the RWC only decreased 0.48%, and this difference was not statistically significant (Fig. 2C). Based on these results, we found that treatment with 10% PEG-6000 for 24 h should be considered as mild osmotic stress (MOS), while treatment with 15% PEG-6000 for 24 h should be considered as severe osmotic stress (SOS).

### Identification of root proteins under osmotic stress using iTRAQ

As roots directly sense osmotic stress, total protein was extracted from the root samples of wheat plants under control, MOS and SOS conditions (two, three and three replicates, respectively). The protein expression profiles of these eight root samples were analyzed in one 8-plex iTRAQ experiment. A total of 150,440 triggered MS/MS spectra were identified, and 2,228 proteins were identified by 7,392 peptides (File S2), and about 45.17% of the identified proteins included at least two unique peptides.

### The DEPs between osmotic stress and control

The 95% confidence interval of each group distribution was constructed to analyze the maximum scope of difference within group, and the results showed that the maximum scope is 0.855–1.17 (File S2). To insure the difference between groups larger than the difference within group, a fold-change of more than 1.2 (more than 1.2 or less than 0.833) was selected as one of the parameters for DEPs selection. To enhance the confidence of DEPs, the following parameters were also considered: each protein with two or more peptides, at least two times differential expression among three repetition and a significance level of *p* < 0.05. Based on these four parameters, a total of 81 DEPs were identified, including 34 DEPs between the MOS and control samples and 64 DEPs between the SOS and control samples. Among these DEPs, 17 DEPs were common in the MOS and SOS samples compared with the control samples, 30 DEPs were down-regulated, and 51 DEPs were up-regulated under osmotic stress.

### Analysis of the DEPs between osmotic stress and control

Among these DEPs, the molecular function information for 69 DEPs was identified. Many proteins had functions in processes, such as carbohydrate metabolism, protein metabolism, phytohormones responsive etc., and the plant protection system played important roles in the wheat roots response to osmotic stress (Tables 1 and 2).

**Table 1:** The differentially expressed proteins between MOS and control.

Protein name	Protein function	Peptides	Coverage	Fold-change between MOS and control	*P*-value
**Protein Metabolism**					
Traes_2BS_7700613D4	40S ribosomal protein	2	22.31	1.24 ± 0.05	0.012
Traes_7AL_65F481DB9	40S ribosomal protein	2	13.82	1.40 ± 0.15	0.044
Traes_5DL_9D4164773	60S ribosomal protein	2	8.20	1.21 ± 0.04	0.010
Traes_1AL_D20D648FD	60S ribosomal protein	4	36.17	1.29 ± 0.11	0.047
Traes_2AL_396E0F5A3	60S ribosomal protein	2	15.03	1.51 ± 0.14	0.025
**Histone Protein**					
Traes_1AS_4CA1A835D1	Histone H2B	4	50.00	1.41 ± 0.09	0.015
Traes_4BL_96E367077	Histone H2A	3	21.33	1.46 ± 0.17	0.042
**CarhohydrateMetabolism**					
Traes_7DS_529BAB150	Sucrose synthase	15	22.00	1.27 ± 0.10	0.040
Traes_3B_BC152C5D7	Glycosyltransferase	2	6.18	1.23 ± 0.05	0.016
Traes_2AL_AEB11A672	Beta-glucosidase	7	15.93	1.26 ± 0.09	0.036
Traes_4AL_8845F411B	UDP-glucose 6-dehydrogenase	12	31.03	0.70 ± 0.07	0.016
Traes_7BS_DE33B2B49	Fructokinase	2	8.79	1.27 ± 0.09	0.037
Traes_4AL_4B09F91AE	Alcohol dehydrogenase	6	24.74	0.77 ± 0.02	0.003
**Phytohormones**					
Traes_4DS_E2055C83D	Abscisic stress-ripening protein	3	56.52	2.53 ± 0.26	0.009
**Antioxidant Protection Proteins**					
Traes_1AL_46245C5D5	Peroxidase	2	42.47	0.79 ± 0.04	0.010
Traes_2BS_9C71D6F5F	Peroxidase	2	7.81	0.80 ± 0.03	0.007
Traes_1DL_7BCE5B151	Glutathione S-transferase	3	20.37	1.60 ± 0.16	0.023
Traes_1AL_CC4CF4E71	Glutathione S-transferase	2	11.86	1.59 ± 0.15	0.020
Traes_4AS_36CB7931F	Glutathione S-transferase	2	14.75	1.38 ± 0.08	0.015
Traes_XX_BEAB3FB5A	Glutathione S-transferase	2	7.12	1.26 ± 0.06	0.018
Traes_6BL_8360C77EF	Glutathione peroxidase	2	16.28	1.21 ± 0.03	0.006
**Other drought resistance proteins**					
Traes_XX_B7AF82F34	ATP synthase subunit beta	6	20.00	1.32 ± 0.08	0.019
Traes_2AL_B7FC2C090	Wali7 protein	3	15.81	1.24 ± 0.01	0.001
Traes_4AL_198AD99FF	Clathrin heavy chain	9	15.15	1.27 ± 0.10	0.045
Traes_1AL_1538AC680	Nucleoside diphosphate kinase	3	11.98	1.27 ± 0.04	0.009
Traes_4BL_B5BF83119	Hemoglobin Hb1	6	46.01	1.58 ± 0.08	0.006
Traes_2DL_47E335BA6	NAD(P)H-dependent 6′-deoxychalcone synthase	2	24.19	1.34 ± 0.10	0.028
Traes_XX_F9BB1AA7A	6-phosphogluconate dehydrogenase	6	21.31	0.79 ± 0.06	0.031
Traes_1BL_BEEBE83B7	Cysteine proteinase inhibitor	2	22.06	0.69 ± 0.02	0.001
Traes_5DL_A6B7B0525	Peptidyl-prolyl cis-trans isomerase	2	16.07	0.81 ± 0.04	0.012
Traes_7AS_8E6B88A80	Pathogenesis-related protein	4	37.27	0.80 ± 0.06	0.033
Traes_2AL_800303D8D	Pathogenesis-related protein	5	42.24	0.80 ± 0.07	0.042
Traes_3DL_3D1319ECF	Acyl-(Acyl-carrier-protein) desaturase	2	10.38	0.58 ± 0.07	0.010
**Uncharacterized proteins**					
Traes_7BL_BE36675C8	Uncharacterized protein	2	8.82	0.74 ± 0.01	0.001

**Table 2:** The differentially expressed proteins between SOS and control.

Protein name	Protein function	Peptides	Coverage	Fold-change between SOS and control	*P*-value
**Protein Metabolism**					
Traes_2BS_7700613D4	40S ribosomal protein	2	22.31	1.25 ± 0.05	0.013
Traes_7AL_65F481DB9	40S ribosomal protein	2	13.82	1.45 ± 0.14	0.031
Traes_3AL_0A1239316	Glycine dehydrogenase	2	5.82	0.83 ± 0.01	0.001
Traes_2DS_64EC7E533	Eukaryotic translation initiation factor 3	3	10.53	0.82 ± 0.07	0.045
Traes_XX_B2924FB2E	Eukaryotic translation initiation factor 3	2	10.71	0.81 ± 0.01	0.001
Traes_4BL_E2E2C4E1D	Adenylate kinase 1	2	13.85	0.81 ± 0.03	0.006
Traes_XX_7DC2CED29	E3 ubiquitin-protein ligase	2	13.75	1.23 ± 0.07	0.031
**Histone Protein**					
Traes_4BL_96E367077	Histone H2A	3	21.33	1.49 ± 0.14	0.024
**CarhohydrateMetabolism**					
Traes_3B_BC152C5D7	Glycosyltransferase	2	6.18	1.38 ± 010	0.021
Traes_4AL_82AB2E772	Beta-fructofuranosidase	7	18.10	0.83 ± 0.02	0.004
Traes_4DS_084803084	Beta-glucosidase	2	5.52	0.80 ± 0.02	0.004
Traes_3B_B8697F82E	Glucan endo-1,3-beta-glucosidase	4	16.87	0.82 ± 0.06	0.034
Traes_4AL_8845F411B	UDP-glucose 6-dehydrogenase	12	31.03	0.73 ± 0.04	0.006
Traes_4BS_11DDF29B31	Xylanase inhibitor protein	2	9.02	0.70 ± 0.06	0.014
Traes_4DL_A80B33149	Beta-amylase	3	10.89	0.74 ± 0.07	0.025
Traes_1DL_FDF182BF9	Hexokinase	6	20.88	1.37 ± 0.07	0.012
Traes_4AL_E6D679339	Alcohol dehydrogenase	3	10.09	1.23 ± 0.08	0.035
Traes_4AL_4B09F91AE	Alcohol dehydrogenase	6	24.74	0.80 ± 0.07	0.041
**Phytohormones**					
Traes_4BS_BB26E5EE1	Abscisic stress-ripening protein	3	56.12	2.39 ± 0.14	0.003
Traes_4DS_E2055C83D	Abscisic stress-ripening protein	3	56.52	2.37 ± 0.24	0.010
**Antioxidant Protection Proteins**					
Traes_2DS_E3F0742FF	Peroxidase	2	24.36	0.82 ± 0.04	0.018
Traes_2DS_2CCCA54C1	Peroxidase	13	56.83	0.78 ± 0.07	0.034
Traes_6AS_621A7A571	Peroxidase	4	25.71	0.78 ± 0.03	0.008
Traes_7DL_D99ED7064	Peroxidase	7	26.39	0.77 ± 0.07	0.032
Traes_2DS_090AF6B73	Peroxidase	3	14.86	0.78 ± 0.01	0.001
Traes_2AL_520618712	Peroxidase	7	24.85	1.20 ± 0.05	0.018
Traes_1DL_7BCE5B151	Glutathione S-transferase	3	20.37	2.04 ± 0.02	0.000
Traes_1AS_D25875432	Glutathione S-transferase	4	20.44	1.32 ± 0.03	0.002
Traes_1AL_CC4CF4E71	Glutathione S-transferase	2	11.86	1.92 ± 0.33	0.040
Traes_1DS_FD8511876	Glutathione S-transferase	5	28.64	1.23 ± 0.08	0.039
Traes_4AS_36CB7931F	Glutathione S-transferase	2	14.75	2.30 ± 0.10	0.002
Traes_6AS_A2A2B273C	Glutathione S-transferase	3	12.55	1.43 ± 0.07	0.009
Traes_1BL_3765A51EC	Glutathione S-transferase	2	10.27	1.50 ± 0.15	0.030
Traes_XX_BEAB3FB5A	Glutathione S-transferase	2	7.12	1.57 ± 0.06	0.004
Traes_1DS_EFDF9CB72	Glutamate-cysteine ligase	5	12.45	1.23 ± 0.01	0.001
Traes_XX_52CBB24F1	glutathione reductase (GR)	6	23.10	1.25 ± 0.03	0.04
Traes_5BL_34593C7D1	Aldehyde oxidase 3	2	2.23	1.29 ± 0.09	0.032
Traes_1AL_5A7E85C4E	Sulfite reductase	6	12.58	1.28 ± 0.10	0.038
Traes_3B_1962330BB	Oxalate oxidase 2	3	33.98	1.40 ± 0.15	0.043
Traes_XX_3D56A9D19	Monodehydroascorbate reductase	3	11.90	0.82 ± 0.01	0.001
** Other drought resistance proteins**					
Traes_5BL_B92355534	Germin-like protein	3	26.41	1.27 ± 0.03	0.004
Traes_4AL_198AD99FF	Clathrin heavy chain	9	15.15	1.28 ± 0.11	0.049
Traes_4BL_B5BF83119	Hemoglobin Hb1	6	46.01	1.89 ± 0.11	0.005
Traes_2DL_47E335BA6	NAD(P)H-dependent 6′-deoxychalcone synthase	2	24.19	1.46 ± 0.12	0.020
Traes_2AL_141C6B5E4	ATP synthase subunit alpha	6	10.59	1.36 ± 0.03	0.003
Traes_XX_175EF4A84	Deoxymugineic acid synthase1	2	10.49	1.28 ± 0.06	0.013
Traes_XX_6A9FEF618	ATP sulfurylase	5	16.17	1.28 ± 0.06	0.016
Traes_5BL_17F1F28B6	Wali7 protein	2	13.08	1.34 ± 0.09	0.024
Traes_XX_F9BB1AA7A	6-phosphogluconate dehydrogenase	6	21.31	0.82 ± 0.03	0.010
Traes_1BL_BEEBE83B7	Cysteine proteinase inhibitor	2	22.06	0.82 ± 0.04	0.017
Traes_4BS_9F3A928B7	Low temperature-responsive RNA-binding protein	2	53.03	0.76 ± 0.03	0.007
Traes_XX_903D8ADBC	Fasciclin-like protein FLA15	2	14.48	0.81 ± 0.04	0.013
**Uncharacterized proteins**					
Traes_7BL_BE36675C8	Uncharacterized protein	2	8.82	0.75 ± 0.02	0.002
Traes_5DL_43046228D	Uncharacterized protein	5	15.97	1.22 ± 0.00	0.000
Traes_3B_67E790B47	Uncharacterized protein	2	3.17	1.20 ± 0.04	0.010
Traes_6BS_4EED05084	Uncharacterized protein	3	14.91	1.74 ± 0.19	0.022
Traes_4AL_6A515079C	Uncharacterized protein	2	9.52	0.78 ± 0.03	0.008
Traes_1BL_BF3813A4B	Uncharacterized protein	2	12.86	1.24 ± 0.02	0.003
Traes_2BL_B09F6D195	Uncharacterized protein	5	11.76	1.22 ± 0.02	0.004
Traes_3B_4490CECAF	Uncharacterized protein	2	6.74	1.21 ± 0.03	0.006
Traes_7AS_C87C2FF27	Uncharacterized protein	3	10.40	1.24 ± 0.05	0.017
Traes_6BS_E96E17B28	Uncharacterized protein	2	30.00	1.47 ± 0.14	0.026
Traes_XX_6DDA59584	Uncharacterized protein	2	5.94	1.34 ± 0.03	0.050
Traes_XX_7F3775F4C	Uncharacterized protein	3	8.97	0.77 ± 0.09	0.048

#### Protein metabolism

Osmotic stress greatly impacted the variety and quantity of expressed proteins in plants. The ribosome is a large complex comprising 40S subunit and 60S subunits, and this complex is responsible for protein synthesis from mRNA. Proteome analysis of the wheat roots found three 60S ribosomal proteins and two 40S ribosomal proteins, which are important components of the ribosome, were up-regulated in the roots under osmotic stress compared with control. In addition, the glycine dehydrogenase, which degrade the glycine, showed down-regulate. These results indicate that the process of translation is more active under osmotic stress in response to the adverse environment.

However, two eukaryotic translation initiation factors, which promote the assembly of the ribosome and initiation code for further translation (You, Coghill & Brown, 2013), were down-regulated, and one E3 ubiquitin-protein ligase, which is involved in ubiquitin mediated proteolysis, was up-regulated under drought stress. These factors do not support functional proteins formation.

Based on the identification of DEPs involved in protein metabolism and the dry weight changes under osmotic stress, we speculated that protein synthesis is more active under osmotic stress, thereby produce a greater variety of proteins to increasing the environmental adaptability in wheat, but the protein quantity is inhibited under osmotic stress.

#### Histone proteins

The nucleosome is the basic unit of chromatin, comprising approximately 147 bp of DNA and a histone octamer composed involving a (Histone3-Histone4)_2_ tetramer and two (Histone2A-Histone2B) dimmers (Luger et al., 1997). The results of the iTRAQ analysis revealed the up-regulation of two histone proteins in the roots under osmotic stresses. This finding indicates a high level of chromatin condensation in the roots under osmotic stress, generating transcriptional inertness and a significant decrease in total protein. It consistent with the results of the dry weight result and protein metabolism analysis, which speculate the protein variety is increased and the protein quantity is inhibited under osmotic stress.

#### Carbohydrate metabolism

Carbohydrates are the primary energy resources for organisms and act as small signaling molecules. Under osmotic stress, water-soluble carbohydrates, such as glucose, fructose, sucrose and fructans, are increased in the stems (Foulkes, Scott & Sylvester-Bradley, 2002; Asseng & Herwaarden, 2003; Ruuska et al., 2006), leaves (Roover et al., 2000) and roots (Roover et al., 2000) to impede water loss in plants. Herein, we also found that the enzymes that catalyze the production of small carbohydrate osmolytes, such as sucrose synthase, glucosidase and glycosyltransferase, were up-regulated except Traes_4DS_084803084 and Traes_3B_B8697F82E, and the enzymes that inhibit the formation of small carbohydrate osmolytes, such as UDP-glucose 6-dehydrogenase and xylanase inhibitor protein, was down-regulated under osmotic stress. This finding indicates that small molecular carbohydrates are produced at significant levels to increase osmotic potential in the roots of wheat under osmotic stress.

Glycolysis is an important metabolic pathway, which would produce energy and carbon skeletons for the primary and secondary metabolites biosynthesis (Cramer et al., 2013). And some previous studies had found that the genes or proteins, involved in glycolysis, would be induced (Rizhsky, Liang & Mittler, 2002; Oh & Komatsu, 2015). In this study, fructokinase, hexokinase and alcohol dehydrogenase, which take part in the pathway of glycolysis, were found to be up-regulated for more energy production under osmotic stress.

#### Phytohormones responsive

Phytohormones play important roles in the adaption of plants to abiotic stresses. The abscisic acid (ABA)-dependent signaling pathway is one of the most important pathways in the resistance to drought stress in plants, and many important drought- or osmotic-related genes, such as AREB1, AREB2, ABF3, SnRK2, and ABF1, are involved in this pathway (Yoshida, Mogami & Yamaguchi-Shinozaki, 2014). In the present study, two abscisic stress-ripening proteins, which can be induced by ABA and abiotic stress (Golan et al., 2014), were up-regulated in the roots under osmotic stress, suggesting that the ABA signaling pathway is important in the resistance of wheat to osmotic stress.

#### Plant protection system

Many studies have demonstrated that ROS and cytotoxin would significantly increase under osmotic conditions, which would induce cellular damage in plants. To prevent the damages, plants have generated many plant protection systems to remove ROS and cytotoxins. Here, we found that the proteins involved in ROS scavenging and detoxifying were up-regulated, except some peroxidase.

**Figure 3:**
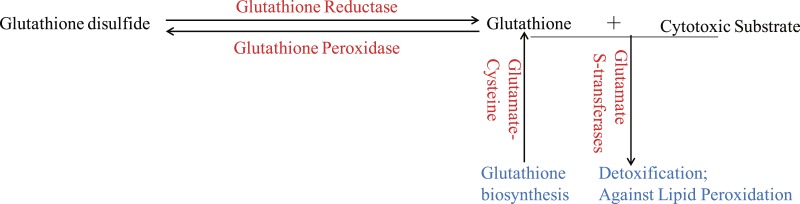
The glutathione system in wheat roots under osmotic stress. The up-regulated proteins were denoted with red color, and the functions of proteins were denoted with blue color.

**Figure 4:**
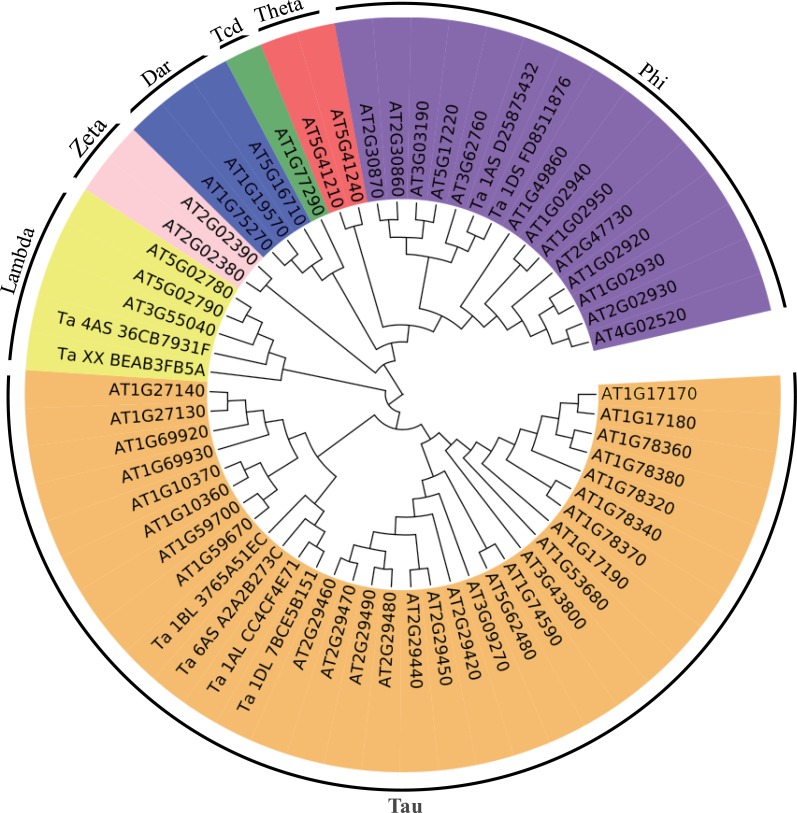
Phylogenetic tree of the GSTs. The unrooted phylogenetic tree of GSTs from *Arabidopsis* and the eight differentially expressed GSTs in the current proteome analysis was constructed by the neighbor-joining method using MEGA 5.10 software. The subgroups of the GSTs are distinguished with different colors.

Glutathione (GSH) has multiple functions, such as antioxidant and detoxification, in plants (Noctor et al., 2012). The ROS would be continuous eliminated by GSH-GSSH (Glutathione disulfide) cycle in organism, which depends on glutathione reductase and glutathione peroxidase. And GSTs, which could be induced through different biotic and abiotic stresses, would protect organisms against oxidative damage and lipid peroxidation, and catalyze the conjugation of electrophilic substrates and glutathione to eliminate cytotoxic substrates (Marrs, 1996; Chen et al., 2012; Yang et al., 2001). In the present study, one glutathione reductase and one glutathione peroxidase were found to be up-regulated (Fig. 3), indicating that GSH-GSSH cycle was more active to maintain ROS balance, under osmotic stress. And eight GSTs were also up-regulated to detoxify harmful materials and maintain cell redox homeostasis in plants under osmotic stress (Fig. 3). In addition, one glutamate-cysteine ligase, which catalyzes the first and rate-limiting step of glutathione biosynthesis, was up-regulated (Fig. 3). All these results showed that glutathione system played important roles in protecting organism from damage caused by osmotic stress in wheat roots.

To better understand the evolutionary relationships of these GSTs, an unrooted phylogenetic tree, including AtGSTs and these eight GSTs, was constructed. We identified two GSTs belonging to the Phi family, two GSTs belonging to the Lambda family and four GSTs belonging to the Tau family (Fig. 4). Most GSTs are Phi or Tau, which are plant-specific GSTs and the major phase II enzymes in a common detoxification pathway (Frova, 2003). Transgenic plants over-expressing Tau or Phi GSTs showed high tolerance to herbicides, salt and UV radiation (Karavangeli et al., 2005; Benekos et al., 2010; Jha, Sharma & Mishra, 2011). These results indicate that glutathione play an important role in the detoxification of cytotoxin under osmotic stress in wheat.

In addition, many other DEPs associated with redox reactions, such as reductase and oxidase, were observed under osmotic stress.

#### Other osmotic resistance proteins

In addition to the proteins mentioned above, twenty DEPs with known functions were also found in this proteome analysis. nine of these DEPs were down-regulated under osmotic stress, including cysteine proteinase inhibitor, adenylate kinase etc. Eleven of these DEPs were up-regulated under osmotic stress, including ATP synthase subunit alpha, Wali7 protein etc.

## Conclusions

In the present study, we used iTRAQ to comprehensively study the protein expression profile in the root of wheat under osmotic stress. A total of 2,228 expressed proteins were identified. Among these, 81 were DEPs associated with protein metabolism, carbohydrate metabolism, phytohormones, plant protection system and other functions. These findings help clarify the response to osmotic stress in wheat and provide additional information for future studies of the mechanism of osmotic resistance in wheat.

##  Supplemental Information

10.7717/peerj.2334/supp-1 File S1 The protein database for iTRAQ analysis

10.7717/peerj.2334/supp-2 File S2 Expressed proteins and the 95% confidence interval between osmotic samples and control

## Additional Information and Declarations

### Data Availability

The following information was supplied regarding data availability: The raw data is provided as Supplemental Files. The mass spectrometry data are deposited in the iProx database (http://www.iprox.cn/index) with the accession number: IPX00075800.

## References

[ref-1] Asseng S, Herwaarden AFV (2003). Analysis of the benefits to wheat yield from assimilates stored prior to grain filling in a range of environments. Plant and Soil.

[ref-2] Benekos K, Kissoudis C, Nianiou-Obeidat I, Labrou N, Madesis P, Kalamaki M, Makris A, Tsaftaris A (2010). Overexpression of a specific soybean *GmGSTU4* isoenzyme improves diphenyl ether and chloroacetanilide herbicide tolerance of transgenic tobacco plants. Journal of Biotechnology.

[ref-3] Caruso G, Cavaliere C, Foglia P, Gubbiotti R, Samperi R, Laganà A (2009). Analysis of drought responsive proteins in wheat (*Triticum durum*) by 2D-PAGEand MALDI-TOF mass spectrometry. Plant Science.

[ref-4] Chen J-H, Jiang H-W, Hsieh E-J, Chen H-Y, Chien C-T, Hsieh H-L, Lin T-P (2012). Drought and salt stress tolerance of an *Arabidopsis* glutathione S-transferase U17 knockout mutant are attributed to the combined effect of glutathione and abscisic acid. Plant Physiology.

[ref-5] Cramer GR, Van Sluyter SC, Hopper DW, Pascovici D, Keighley T, Haynes PA (2013). Proteomic analysis indicates massive changes in metabolism prior to the inhibition of growth and photosynthesis of grapevine (*Vitis vinifera* L.) in response to water deficit. BMC Plant Biology.

[ref-6] Deeba F, Pandey AK, Ranjan S, Mishra A, Singh R, Sharma YK, Shirke PA, Pandey V (2012). Physiological and proteomic responses of cotton (*Gossypium herbaceum* L.) to drought stress. Plant Physiology and Biochemistry.

[ref-7] Ding Z, Li S, An X, Liu X, Qin H, Wang D (2009). Transgenic expression of *MYB15* confers enhanced sensitivity to abscisic acid and improved drought tolerance in *Arabidopsis thaliana*. Journal of Genetics and Genomics.

[ref-8] Fan J, Chen C, Yu Q, Brlansky RH, Li Z-G, Gmitter FG (2011). Comparative iTRAQ proteome and transcriptome analyses of sweet orange infected by “*Candidatus Liberibacter asiaticus*”. Physiologia Plantarum.

[ref-9] Foulkes MJ, Scott TLRK, Sylvester-Bradley R (2002). The ability of wheat cultivars to withstand drought in UK conditions: formation of grain yield. The Journal of Agricultural Science.

[ref-10] Frova C (2003). The plant glutathione transferase gene family: genomic structure, functions, expression and evolution. Physiologia Plantarum.

[ref-11] Gao L, Yan X, Li X, Guo G, Hu Y, Ma W, Yan Y (2011). Proteome analysis of wheat leaf under salt stress by two-dimensional difference gel electrophoresis (2D-DIGE). Phytochemistry.

[ref-12] Ge P, Ma C, Wang S, Gao L, Li X, Guo G, Ma W, Yan Y (2012). Comparative proteomic analysis of grain development in two spring wheat varieties under drought stress. Analytical and Bioanalytical Chemistry.

[ref-13] Ge X, Zhang C, Wang Q, Yang Z, Wang Y, Zhang X, Wu Z, Hou Y, Wu J, Li F (2014). iTRAQ protein profile differential analysis between somatic globular and cotyledonary embryos reveals stress, hormone, and respiration involved in increasing plantlet regeneration of *Gossypium hirsutum* L. Journal of Proteome Research.

[ref-14] Golan I, Dominguez PG, Konrad Z, Shkolnik-Inbar D, Carrari F, Bar-Zvi D (2014). Tomato *ABSCISIC ACID STRESS RIPENING* (*ASR*) gene family revisited. PLoS ONE.

[ref-15] Hu H, Dai M, Yao J, Xiao B, Li X, Zhang Q, Xiong L (2006). Overexpressing a NAM, ATAF, and CUC (NAC) transcription factor enhances drought resistance and salt tolerance in rice. Proceedings of the National Academy of Sciences of the United States of America.

[ref-16] Jha B, Sharma A, Mishra A (2011). Expression of *SbGSTU* (tau class glutathione S-transferase) gene isolated from* Salicornia brachiata* in tobacco for salt tolerance. Molecular Biology Reports.

[ref-17] Karavangeli M, Labrou NE, Clonis YD, Tsaftaris A (2005). Development of transgenic tobacco plants overexpressing maize glutathione S-transferase I for chloroacetanilide herbicides phytoremediation. Biomolecular Engineering.

[ref-18] Krasensky J, Jonak C (2012). Drought, salt, and temperature stress-induced metabolic rearrangements and regulatory networks. Journal of Experimental Botany.

[ref-19] Lan P, Li W, Wen T-N, Shiau J-Y, Wu Y-C, Lin W, Schmidt W (2011). iTRAQ protein profile analysis of *Arabidopsis* roots reveals new aspects critical for iron homeostasis. Plant Physiology.

[ref-20] Le DT, Nishiyama R, Watanabe Y, Tanaka M, Seki M, Ham le H, Yamaguchi-Shinozaki K, Shinozaki K, Tran LS (2012). Differential gene expression in soybean leaf tissues at late developmental stages under drought stress revealed by genome-wide transcriptome analysis. PLoS ONE.

[ref-21] Lenka SK, Katiyar A, Chinnusamy V, Bansal KC (2011). Comparative analysis of drought-responsive transcriptome in Indica rice genotypes with contrasting drought tolerance. Plant Biotechnology Journal.

[ref-22] Li C, Li T, Zhang D, Jiang L, Shao Y (2013). Exogenous nitric oxide effect on fructan accumulation and FBEs expression in chilling-sensitive and chilling-resistant wheat. Environmental and Experimental Botany.

[ref-23] Li Y, Meng F, Zhang C, Zhang N, Sun M, Ren J, Niu H, Wang X, Yin J (2012). Comparative analysis of water stress-responsive transcriptomes in drought-susceptible and -tolerant wheat (*Triticum aestivum* L.). Journal of Plant Biology.

[ref-24] Liu Q, Kasuga M, Sakuma Y, Abea H, Miuraa S, Yamaguchi-Shinozakia K, Shinozakib K (1998). Two transcription factors, *DREB1* and *DREB2*, with an EREBP/AP2 DNA binding domain separate two cellular signal transduction pathways in drought- and low-temperature-responsive gene expression, respectively, in *Arabidopsis*. The Plant Cell.

[ref-25] Liu G, Li X, Jin S, Liu X, Zhu L, Nie Y, Zhang X (2014). Overexpression of rice NAC gene *SNAC1* improves drought and salt tolerance by enhancing root development and reducing transpiration rate in transgenic cotton. PLoS ONE.

[ref-26] Longworth J, Noirel J, Pandhal J, Wright PC, Vaidyanathan S (2012). HILIC-and SCX-based quantitative proteomics of Chlamydomonas reinhardtii during nitrogen starvation induced lipid and carbohydrate accumulation. Journal of Proteome Research.

[ref-27] Luger K, Mäder AW, Richmond RK, Sargent DF, Richmond TJ (1997). Crystal structure of the nucleosome core particle at 2.8 Å resolution. Nature.

[ref-28] Ma J, Zhang D, Shao Y, Liu P, Jiang L, Li C (2014). Genome-wide analysis of the WRKY transcription factors in *Aegilops tauschii*. Cytogenetic and Genome Research.

[ref-29] Marrs KA (1996). The functions and regulation of glutathione S-transferases in plants. Annual Review of Plant Physiology and Plant Molecular Biology.

[ref-30] Matsui A, Ishida J, Morosawa T, Mochizuki Y, Kaminuma E, Endo TA, Okamoto M, Nambara E, Nakajima M, Kawashima M, Satou M, Kim JM, Kobayashi N, Toyoda T, Shinozaki K, Seki M (2008). *Arabidopsis* transcriptome analysis under drought, cold, high-salinity and ABA treatment conditions using a tiling array. Plant and Cell Physiology.

[ref-31] Mayer KFX, Rogers J, Doležel J, Pozniak C, Eversole K, Feuillet C, Gill B, Friebe B, Lukaszewski AJ, Sourdille P, Endo TR, Kubaláková M, Cíhalíková J, Dubská Z, Vrána J, Sperková R, Simková H, Febrer M, Clissold L, McLay K, Singh K, Chhuneja P, Singh NK, Khurana J, Akhunov E, Choulet F, Alberti A, Barbe V, Wincker P, Kanamori H, Kobayashi F, Itoh T, Matsumoto T, Sakai H, Tanaka T, Wu J, Ogihara Y, Handa H, Maclachlan PR, Sharpe A, Klassen D, Edwards D, Batley J, Olsen OA, Sandve SR, Lien S, Steuernagel B, Wulff B, Caccamo M, Ayling S, Ramirez-Gonzalez RH, Clavijo BJ, Wright J, Pfeifer M, Spannagl M, Martis MM, Mascher M, Chapman J, Poland JA, Scholz U, Barry K, Waugh R, Rokhsar DS, Muehlbauer GJ, Stein N, Gundlach H, Zytnicki M, Jamilloux V, Quesneville H, Wicker T, Faccioli P, Colaiacovo M, Stanca AM, Budak H, Cattivelli L, Glover N, Pingault L, Paux E, Sharma S, Appels R, Bellgard M, Chapman B, Nussbaumer T, Bader KC, Rimbert H, Wang S, Knox R, Kilian A, Alaux M, Alfama F, Couderc L, Guilhot N, Viseux C, Loaec M, Keller B, Praud S (2014). A chromosome-based draft sequence of the hexaploid bread wheat (*Triticum aestivum*) genome. Science.

[ref-32] Mirzaei M, Soltani N, Sarhadi E, Pascovici D, Keighley T, Salekdeh GH, Haynes PA, Atwell BJ (2012). Shotgun proteomic analysis of long-distance drought signaling in rice roots. Journal of Proteome Research.

[ref-33] Nir I, Moshelion M, Weiss D (2014). The *Arabidopsis GIBBERELLIN METHYL TRANSFERASE 1* suppresses gibberellin activity, reduces whole-plant transpiration and promotes drought tolerance in transgenic tomato. Plant, Cell and Environment.

[ref-34] Noctor G, Mhamdi A, Chaouch S, Han Y, Neukermans J, Marquez-Garcia B, Queval G, Foyer CH (2012). Glutathione in plants: an integrated overview. Plant, Cell and Environment.

[ref-35] Oh MW, Komatsu S (2015). Characterization of proteins in soybean roots under flooding and drought stresses. Journal of Proteomics.

[ref-36] Pasquali G, Biricolti S, Locatelli F, Baldoni E, Mattana M (2008). *Osmyb4* expression improves adaptive responses to drought and cold stress in transgenic apples. Plant Cell Reports.

[ref-37] Peng Z, Wang M, Li F, Lv H, Li C, Xia G (2009). A proteomic study of the response to salinity and drought stress in an introgression strain of bread wheat. Molecular & Cellular Proteomics.

[ref-38] Qin X, Zeevaart JAD (2002). Overexpression of a 9-cis-epoxycarotenoid dioxygenase gene in *Nicotiana plumbaginifolia* increases abscisic acid and phaseic acid levels and enhances drought tolerance. Plant Physiology.

[ref-39] Qiu Y, Yu D (2009). Over-expression of the stress-induced *OsWRKY45* enhances disease resistance and drought tolerance in *Arabidopsis*. Environmental and Experimental Botany.

[ref-40] Rizhsky L, Liang H, Mittler R (2002). The combined effect of drought stress and heat shock on gene expression in tobacco. Plant Physiology.

[ref-41] Roover JD, Vandenbranden K, Laere AV, Ende WVD (2000). Drought induces fructan synthesis and 1-SST (sucrose: sucrose fructosyltransferase) in roots and leaves of chicory seedlings (*Cichorium intybus* L.). Planta.

[ref-42] Ruuska SA, Rebetzke GJ, Herwaarden AFV, Richards RA, Fettell NA, Tabe L, Jenkins CLD (2006). Genotypic variation in water-soluble carbohydrate accumulation in wheat. Functional Plant Biology.

[ref-43] Shinozaki K, Yamaguchi-Shinozakiy K, Sekiz M (2003). Regulatory network of gene expression in the drought and cold stress responses. Current Opinion in Plant Biology.

[ref-44] Shou H, Bordallo P, Wang K (2004). Expression of the Nicotiana protein kinase (*NPK1*) enhanced drought tolerance in transgenic maize. Journal of Experimental Botany.

[ref-45] Valliyodan B, Nguyen HT (2006). Understanding regulatory networks and engineering for enhanced drought tolerance in plants. Current Opinion in Plant Biology.

[ref-46] Yang Y, Cheng JZ, Singhal SS, Saini M, Pandya U, Awasthi S, Awasth YC (2001). Role of glutathione S-transferases in protection against lipid peroxidation. Journal of Biological Chemistry.

[ref-47] Yoshida T, Mogami J, Yamaguchi-Shinozaki K (2014). ABA-dependent and ABA-independent signaling in response to osmotic stress in plants. Current Opinion in Plant Biology.

[ref-48] You T, Coghill GM, Brown AJP (2013). Eukaryotic translation initiation factor interactions. Encyclopedia of systems biology.

[ref-49] Zheng J, Fu J, Gou M, Huai J, Liu Y, Jian M, Huang Q, Guo X, Dong Z, Wang H, Wang G (2010). Genome-wide transcriptome analysis of two maize inbred lines under drought stress. Plant Molecular Biology.

[ref-50] Zhu JK, Hasegawa PM, Bressan RA, Bohnert HJ (1997). Molecular aspects of osmotic stress in plants. Critical Reviews in Plant Sciences.

